# Improvement of Albendazole Bioavailability with Menbutone Administration in Sheep

**DOI:** 10.3390/ani12040463

**Published:** 2022-02-14

**Authors:** Raquel Diez, M. Jose Diez, Juan J. Garcia, Jose M. Rodríguez, Cristina Lopez, Nelida Fernandez, Matilde Sierra, Ana M. Sahagun

**Affiliations:** Pharmacology, Department of Biomedical Sciences, Veterinary Faculty, Institute of Biomedicine (IBIOMED), University of Leon, 24071 Leon, Spain; rdielz@unileon.es (R.D.); mjdiel@unileon.es (M.J.D.); jjgarv@unileon.es (J.J.G.); jmrodl@unileon.es (J.M.R.); mnferm@unileon.es (N.F.); msiev@unileon.es (M.S.)

**Keywords:** albendazole, albendazole sulfoxide, albendazole sulfone, bioavailability, interaction, menbutone, pharmacokinetics, sheep

## Abstract

**Simple Summary:**

Anthelmintic drugs are among those most widely used in veterinary practice. The development of resistance to these drugs is a widespread problem, especially in small ruminants, and represents a serious threat to animal production. Thus, new possibilities to use the available pharmacological groups in a more efficient way should be explored. The objective of this study was to assess the pharmacokinetic interaction between a benzimidazole (albendazole, ABZ) and a choleretic drug (menbutone, MEN) in sheep, and it may result in greater effectivity of this drug against nematode parasites. Plasma concentrations of ABZSO (ABZ active metabolite) were higher when ABZ was administered with MEN. The proposed interaction is a simple, safe, and inexpensive way of increasing the effectivity of this anthelmintic widely used in livestock.

**Abstract:**

The pharmacokinetic interaction between a benzimidazole (albendazole, ABZ) and a choleretic drug (menbutone, MEN) was evaluated in sheep. The plasma disposition of albendazole sulfoxide (ABZSO, active metabolite) and albendazole sulfone (ABZSO_2_, inactive metabolite) was investigated following an oral administration of albendazole (ABZ) (5 mg/kg) alone or with menbutone (MEN) (intramuscular, 10 mg/kg). Blood samples were collected over 3 days post-treatment, and drug plasma concentrations were measured by high performance liquid chromatography (HPLC). ABZSO was measured from 0.5 to 48 h, and ABZSO_2_ from 2 to 60 h. No parent drug was detected at any sampling time. Mean maximum plasma concentration (C_max_) and the area under the plasma concentration-time curve (AUC) were 12.8% and 21.5% higher for ABZSO when ABZ and MEN were administered together, which indicates a significant increase in the amount absorbed. The rate of absorption was not modified, with similar values for the time to reach C_max_ (t_max_) (11.5 h with ABZ + MEN and 10.7 h with ABZ treatment), although no significant differences were observed for these latter pharmacokinetic parameters. Regarding ABZSO_2_, C_max_, AUC and t_max_ values were similar after both treatments (ABZ or ABZ + MEN). The results obtained indicate that co-administration of ABZ and MEN may be an interesting and practical option to increase the efficacy of this anthelmintic.

## 1. Introduction

Albendazole (ABZ) is one of the most extensively used anthelmintic drugs in ruminants as it exhibits high efficacy against a broad spectrum of gastrointestinal and bronchopulmonary worms, little toxicity, and low cost. As with other benzimidazoles, its low aqueous solubility is a major disadvantage, since ABZ is poorly absorbed after oral administration, which limits its clinical use [1,2]. Benzimidazoles bind to parasite β-tubulin, necessary for the formation of microtubules, inhibiting all microtubule-based processes in the parasites, which leads to worm death [3,4].

ABZ plasma disposition has been widely studied in different animal species, including ruminants [5,6,7,8,9,10,11,12,13,14,15]. In these latter animal species, the drug undergoes a complex and extensive biotransformation. After oral administration, the parent drug is absorbed in the small intestine and rapidly and extensively metabolized in the liver through a two-step oxidative reaction into albendazole sulphoxide (ABZSO), the main responsible for the anthelmintic activity, and albendazole sulfone (ABZSO_2_), an inactive compound. ABZSO is also reduced back to ABZ by ruminal and intestinal microflora [16,17,18,19,20]. Moreover, the rumen acts as a drug reservoir, being released slowly into the abomasum, where the acidic pH improves its dissolution.

The development of resistance to the available classes of anthelmintic drugs is a widespread problem, especially in small ruminants, and represents a serious threat to animal production. A rational use of ABZ, based on a better knowledge of its pharmacological properties together with appropriate management strategies in livestock, would be a feasible approach to maintain its therapeutic potential and protect animal health.

The lack of new pharmacological groups to treat parasitosis in veterinary medicine means that the currently available groups should be used in a much more efficient way. The rate of dissolution in the gastrointestinal tract of benzimidazoles is thought to be critical in achieving adequate absorption and, consequently, clinical efficacy. Different approaches have been assessed to increase its oral bioavailability, including alternative drug formulations to improve aqueous solubility [2,21], interferences in liver biotransformation [15,22], and non-chemical strategies, such as management of feed intake [23,24] or prior fasting to administration [25,26]. Although these approaches have improved the knowledge on the pharmacokinetic behavior of ABZ, none of them has been finally translated into the SPC of the medicinal products containing ABZ and thus, to daily clinical practice. 

Previous studies have shown that the surfactant effect of the bile salts may be related to a higher dissolution of ABZ in the gastrointestinal tract and, consequently, to an increased absorption of this drug [27,28,29]. Solubility, but not absorption, was the rate limiting step in the bioavailability of this anthelmintic [30,31].

In this context, we hypothesize that ABZ oral bioavailability may be modified by increasing its solubility and intestinal absorption with the coadministration of menbutone (MEN), a choleretic drug commonly used in sheep. MEN, also known as genabilic acid, increases bile, pancreatic, and peptic secretions by 2–5 times baseline. The effect is observed in a few minutes after administration, and remains for 2–3 h [32,33]. Menbutone has been used in veterinary practice for years to treat digestive disorders. This compound is indicated to stimulate hepato-digestive activity in case of digestive disorders and hepatic insufficiency in sheep, cattle, and goats. In the last decade, several veterinary medicines containing this active ingredient have been approved for use in most EU countries (by national authorization or mutual-recognition procedures) [34,35].

Thus, the present study was carried out to evaluate the potential in vivo drug–drug interaction after the co-administration of ABZ and MEN in sheep, and to assess if MEN could affect the pharmacokinetic profile of this anthelmintic and its metabolites. The plasma concentrations of ABZ, ABZSO, and ABZSO_2_ were evaluated after administration of albendazole, either alone or concomitantly with menbutone.

## 2. Materials and Methods

### 2.1. Animals, Experimental Design and Sampling Procedures

Twelve healthy adult (4–5 years old) non-pregnant and non-lactating female Churra sheep, weighing 50–55 kg, were used. They were allocated in the Experimental Farm of the University of Leon. Sheep were housed indoors in an adequately ventilated building (temperature 19 ± 2 °C). They were allocated for 15 days before the trial began to allow them to acclimate to their environment and maintained in these housing conditions until the end of the experiment. Animals’ health was closely monitored before and throughout the experimental period by a veterinarian. They were maintained on a diet of hay and pelleted feed concentrate twice a day, with water and saltlick ad libitum.

Commercially available formulations of ABZ (Sinvermin ovino^®^, Laboratorios Syva S.A.U., Leon, Spain) and MEN (Digestosyva^®^, Laboratorios Syva S.A.U., Leon, Spain) were administered to animals. A randomized 2-period crossover design was carried out. Sheep were divided into two groups of six animals each. Group 1 received first ABZ orally at a dose of 5 mg/kg. The drug was administered as oral suspension with a dosing device. After a 2-week washout period, menbutone (MEN) (10 mg/kg) was administered into the deep gluteal muscle of the right hind limb immediately after oral ABZ (5 mg/kg). In group 2, animals received concomitant ABZ (oral) and MEN (intramuscular) treatment first and, after a 2-week washout period, oral ABZ, at the same doses previously described.

In both groups, blood samples were collected by venipuncture from both jugular veins into heparinized tubes (Vacutainer^®^, BD, Plymouth, UK) just prior to administration (0) and at 0.25, 0.5, 1, 2, 4, 6, 8, 10, 12, 18, 24, 30, 36, 48, 60, and 72 h thereafter. Samples were immediately centrifuged at 1500 rpm for 20 min, and the recovered plasma stored at −20 °C until analysis.

Animal procedures and management protocols were authorized in advance by both the Ethics Committee of the University of Leon and the regional authorities (ULE-007-2020). No invasive procedure was involved beyond blood sampling.

### 2.2. Reagents

Pure reference standards of ABZ, ABZSO, ABZSO_2_, and oxibendazol (internal standard, IS) were purchased from Sigma-Aldrich (Merck, Darmstadt, Germany). Acetonitrile and methanol solvents used for drug extraction and analysis were HPLC grade, and obtained from Merck (Darmstadt, Germany). Ultrapure water was produced in our laboratory by using a Millipore Milli-Q Gradient water purification system (Waters Corporation, Mildford, MA, USA).

### 2.3. Analytical Procedures

Extraction of ABZ and metabolites (ABZSO and ABZSO_2_) from plasma samples was performed within a maximum of 6–8 weeks. Plasma samples (1 mL) were spiked with 40 µg IS (25 µg/mL). Experimental and spiked plasma samples were analyzed by high-performance liquid chromatography (HPLC) according to a method previously validated [30].

A solid-phase extraction (SPE) procedure was carried out to extract ABZ and metabolites from plasma samples. Cartridges (Oasis HLB 1 cc 30 mg, Waters Corporation, Mildford, MA, USA) were conditioned with 1 mL of methanol and 1 mL of water. Then, 1 mL of plasma was added, and cartridges were washed with 3 mL of water, dried with air for 5 min, and eluted with 2 mL of methanol. The eluate was evaporated to dryness under nitrogen stream. The residue was then reconstituted with 0.25 mL of mobile phase, and 50 µL were injected into the chromatographic system.

Samples were analyzed for ABZ and metabolites in a HPLC system Waters Alliance e2695 equipped with photodiode array detector (model 2998) (Waters Corporation, Mildford, MA, USA). Chromatographic separation was performed by using an XBridge C_18_ column (4.6 mm × 250 mm internal diameter, 5 µm, Waters). The PDA detector was set up at 292 nm. The mobile phase (acetonitrile: ammonium acetate buffer 0.025 M, pH = 6.6) was pumped with variable gradient during the run (12 min). Gradient elution changed from 27:73 to 50:50 in 5 min, maintaining it for 4 min, and returning to 27:73 in 1 min, in which was maintained for 2 min. Flow rate was 1.2 mL/min and injection volume 50 µL. In these conditions, retention times were 8.5 min for ABZ, 3.5 min for ABZSO, 4.9 min for ABZSO_2_, and 7.0 min for oxibendazole (IS). Compounds were identified by comparing with the retention times of pure reference standards. The study was conducted under the Good Laboratory Practice (GLP) regulations at our GLP-compliant laboratory LAFARLE (University of Leon, Spain), certified by the Spanish Agency of Medicines and Medical Devices (AEMPS) [36].

The following parameters were established according to European Medicines Agency guideline EMA/CHMP/EWP/192217/2009: selectivity, lower limit of quantification (LLOQ), calibration curve, accuracy, precision, and stability [37].

Calibration curves (0.025 to 2 µg/mL) were linear for each analyte, with correlation coefficients in the range of 0.995 to 0.999. Recovery percentages were 100.1%, 99.5%, and 95% for ABZSO, ABZSO_2_, and ABZ, respectively. The limits of quantification (LLOQ) were 0.025 µg/mL for the three compounds. No interferences among analytes were observed under current chromatographic conditions. ABZ and their metabolites in working standard solutions and samples were stable for all temperatures and times analyzed (at room temperature for 24 h, at 4–8 °C for 48 h, and at −20 °C for 7 days), with CV always <15% of the nominal concentration (see Figure S1 and Tables S1 and S2, supplementary file).

### 2.4. Pharmacokinetic Analysis

The pharmacokinetic parameters of albendazole for each sheep individually were performed by non-compartmental analysis using Phoenix WinNonLin 8.3 (Certara, Princeton, NJ, USA), with expressions based on statistical moments theory [38] and standard formulae [39,40]. The elimination rate constant (λ), terminal elimination half-life (t_1/2λ_), area under the curve (AUC), area under the moment curve (AUMC), mean residence time (MRT), and the time of observation prior to the first observation with a measurable concentration (t_lag_) were calculated. Plasma elimination rate constant (λ) was estimated by least squares regression of the logarithm of plasma concentration versus time curve over the terminal elimination phase, and t_1/2λ_ as 0.693/λ. AUC and AUMC were calculated by the trapezoidal rule from the time of treatment administration to the last measurable concentration, and further extrapolated to infinity by dividing the last experimental concentration by the terminal slope (λ). MRT was calculated as AUMC/AUC. Maximum plasma concentration (C_max_) and the time to reach C_max_ (t_max_) were determined by direct observation of the plasma concentration-time curves.

### 2.5. Statistical Analysis

Pharmacokinetic parameters were calculated for each sheep and reported as mean ± standard deviation (SD). The statistical analysis was performed using the IBM SPSS for Windows software package v. 26 (IBM Corporation, Armonk, NY, USA). Shapiro–Wilk test was used to test for normality. If data were normal, a paired *t* test was used to evaluate differences between data sets; if not, a Wilcoxon signed-rank test was used. Values were considered significantly different at *p* ≤ 0.05.

## 3. Results

No adverse response was observed in animals for any of the treatments during the study. The parent drug was not detected in any plasma sample after ABZ oral administration to sheep, and only its metabolites (ABZSO and ABZSO_2_) were recovered from samples.

**Figure 1:**
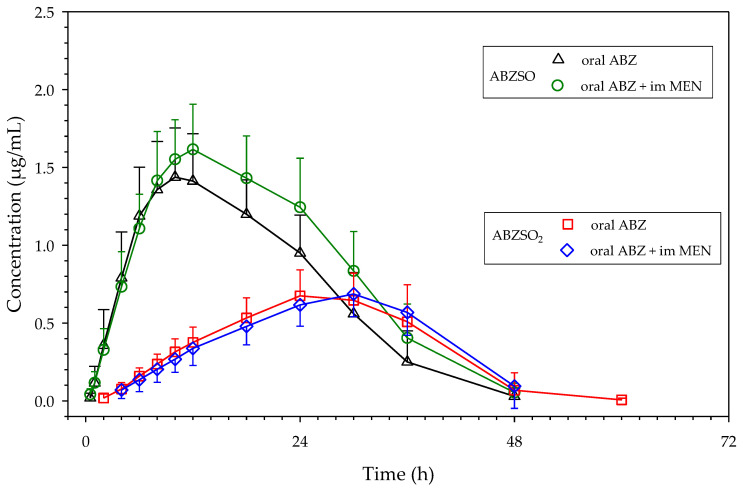
Plasma concentrations (mean ± SD) of ABZSO and ABZSO_2_ obtained after oral ABZ administration (5 mg/kg), and oral ABZ (5 mg/kg) + intramuscular MEN (10 mg/kg) administration to 12 sheep.

Mean and individual plasma concentrations of ABZSO and ABZSO_2_ as a function of time are shown in Figure 1 and Figure S2, respectively, as well as in Tables S3–S6 (see supplementary file).

ABZSO (active metabolite) was detected in plasma between 0.5 and 48 h after ABZ or ABZ + MEN administrations. ABZSO_2_ (inactive metabolite) was present for a longer time when ABZ was administered alone (2–60 h) than after ABZ + MEN administration (4–48 h). ABZSO mean concentrations were always higher than those detected for ABSZO_2_ until 30 h, falling below those values determined for the inactive metabolite from this sampling time onwards. This trend is also observed in most of the individual plasma concentration-time curves.

ABZSO displayed similar plasma concentration profiles when the parent drug was administered alone or concomitantly with MEN, but higher concentrations were achieved when both drugs (anthelmintic and choleretic) were associated, as shown in mean and most individual plasma concentration-time curves (Figure 1 and Figure S2).

The mean non-compartmental pharmacokinetic parameters calculated for ABZSO after administration of ABZ or ABZ + MEN are summarized in Table 1. There were significant differences in the amount of ABZSO generated following the administration of ABZ + MEN versus ABZ alone. When the parent compound was administered with the choleretic drug, C_max_ increased significantly (1.48 µg/mL with ABZ alone vs. 1.67 µg/mL with ABZ + MEN). This concomitant administration also produced significantly higher values of AUC_last_ (34.0 µg·h/mL with ABZ alone vs. 41.3 µg·h/mL with ABZ + MEN). The same behavior was observed in most of the animals, C_max_ increased in 10 animals, diminished in 1 animal, and did not change in 1 sheep; and AUC_last_ increased in 9 animals, and was lower in 3 sheep.

Regarding absorption rate, it did not change as no significant differences were observed in t_max_ mean values (10.7 h vs. 11.5 h). The same situation was also observed when individual plasma concentration–time curves were considered, in which the same number of animals showed a slight increase or decrease in t_max_ values.

For ABZSO_2_ (inactive metabolite), plasma concentration profiles were similar when ABZ was administered alone or with MEN (Figure 1), and no significant differences were observed for C_max_, AUC_last_, and t_max_ (Table 2). Interindividual variations can be seen in Figure S2: in approximately half of the animals, there is an increase, and in the other half, a decrease in both C_max_ and AUC_last_. t_max_ values remain unchanged in most animals, with slight increases or decreases in the others.

## 4. Discussion

ABZ was not detected in any plasma sample. Its extensive metabolization to ABZSO and ABZSO_2_, and the plasma profiles obtained in this study for both metabolites after oral administration to sheep are consistent with those previously published in other studies [11,14,21,41,42,43,44,45].

After MEN administration, plasma concentration profiles and pharmacokinetic parameters obtained for the active metabolite (ABZSO) indicate that the amount absorbed increases significantly (C_max_ and AUC_last_) without modifying its rate (t_max_). MEN increased ABZSO C_max_ by 12.8% and AUC_last_ by 21.5%.

Pharmacokinetic parameters obtained in our study are compared only with those indicated by other authors after ABZ administration by the oral route to sheep at the same dose (5 mg/kg), as a lack of proportionality was described for some ABZSO parameters (C_max_ and AUC), with increasing dosage in this animal species [45].

Regarding ABZSO pharmacokinetic parameters, mean values calculated in this study for AUC_last_ after ABZ administration alone were slightly higher than that reported in adult animals (32.7 µg·h/mL) [46], and larger than those obtained in lambs (23.2–24.2 µg·h/mL) [9]. C_max_ values are between those calculated by other authors (1.30–2.03 µg/mL) [43,46,47] in adult sheep, but higher than in lambs (1.27–1.35 µg/mL) [9,47,48]. As for t_max_, our values were always within the range indicated by other authors in both adult sheep and lambs (8–12.5 h) [9,43,46,47]. ABZSO appeared before in plasma in our study, with a t_lag_ (0.27 h) shorter than that indicated by Lanusse et al. [43] in adult sheep (0.8 h) after ABZ administration. As for MRT_last_, our values were also similar to those calculated in this animal species (13.2–18.1 h) [9,46].

The greater amount of ABZSO after ABZ + MEN administration appeared to be related to a higher absorption of ABZ, and not due to a delayed elimination, as MRT_0–__∞_ and elimination half-life were not significantly different from values obtained when ABZ was administered alone. C_max_ significantly increased by 12.8% when MEN was co-administered with ABZ, although further studies should be developed to confirm its clinical significance.

MEN may positively affect the absorption of ABZSO from the intestinal lumen due to its choleretic properties, increasing bile secretion to intestine and thus, the extent of ABZSO absorbed due to its surfactant capacity. On the other hand, taking into account that this metabolite undergoes enterohepatic recirculation, it would lead to greater and sustained intestinal and plasma concentrations of ABZSO. These higher levels will improve its antihelmintic activity against gastrointestinal worms, where some of the most pathogenic parasites of ruminants are located [49,50], and against lung helminths, as diffusion through external surface is the main mechanism of drug entry to parasites.

Regarding ABZSO_2_, as mentioned above, its concentration-time pattern is also similar to those obtained in previous studies [11,21,25,41,42,43]. In our study, ABZSO_2_ pharmacokinetic parameters show no significant variation in either the amount or the rate of incorporation of this metabolite into the blood.

AUC_last_ mean value calculated in this study was clearly larger as those indicated in both adult sheep and lambs (8.6–10.5 µg·h/mL) [9,46]. In the same way, C_max_ was also higher than those obtained in adult sheep (0.42–0.56 µg/mL) [43,46] and lambs (0.35–0.48 µg/mL) [9,47,48]. t_max_ was also longer in our study than in others carried out in adult sheep (15–24 h) [43,46] and similar to that observed in lambs (30 h) [48]. Mean t_lag_ (2.0 h) for ABZSO_2_ was less than half of that indicated by Lanusse et al. [43] in adult sheep (4.9 h) after ABZ administration. Our MRT values were similar to that obtained in adult sheep (25.3 h) [46], and longer than in lambs (18.6–19.9 h) [9].

At present there is a need to find new pharmacological tools to ensure an efficient control of parasites in domestic animals. Assessment of drug interactions are increasing in recent years to explore their possibilities as a way to control parasites in livestock. Manipulation of ABZ pharmacokinetic behavior to extend its systemic availability has already assayed with variable results. Coadministration of thymol with ABZ has revealed a negative pharmacokinetic interaction in sheep [51]. Other authors have reported higher values of AUC and C_max_ than ours for ABZSO when piperonyl butoxide was dosed with ABZ [15]. Nevertheless, in this latter interaction, other factors, such as the toxicity for the host or the cost of treatment, limited the use of this potential combination [15]. Other strategies, designed to improve ABZ dissolution rate have not been introduced into veterinary clinical practice to date.

Little information about the influence of tensoactive agents on the pharmacokinetics of ABZ is available. A higher gastrointestinal absorption of ABZ was documented with surfactants in rats [52,53] and cattle for sodium lauryl sulphate [7]. However, these latter authors did not find that absorption improved with sodium taurocholate. Surfactants may augment the permeability of biological membranes and enhance the dissolution rate of ABZ. MEN could participate in the mechanisms proposed. More recently, Ochoa et al. [28] reported that a fatty meal enhances ABZ oral bioavailability in healthy human subjects, attributing this higher absorption to food-induced stimulation of bile secretion, increasing the drug solubility.

MEN is a compound indicated as choleretic and, thus, stimulates the function of digestive tract. Regarding its safety, the EU has established that no MRL is required for this drug in food-producing animals [54].

Although the commercial formulation used in this study already contained surfactant agents, MEN improved the absorption of ABZ, with significant increases in C_max_ and AUC_last_ for ABZSO. The results obtained here show that MEN, when administered with ABZ, was able to increase plasma concentrations of ABZSO and, consequently, its anthelmintic effect, but we have evaluated the interaction between ABZ and MEN with the lowest dose (5 mg/kg). It would be interesting to assess if a proportional increase in concentrations would be achieved when the highest dose of ABZ is assayed (7.5 mg/kg).

## 5. Conclusions

The pharmacokinetic behavior of albendazole metabolites was characterized after oral administration of the anthelmintic albendazole (5 mg/kg) with the choleretic agent menbutone (intramuscular, 10 mg/kg) in sheep. We demonstrated that menbutone augmented the amount in blood of ABZSO (active metabolite), significantly increasing C_max_ and AUC_last_, which may contribute to a higher anthelmintic activity in this ruminant species. The association proposed is a simple, safe, and inexpensive way of increasing the effectivity of this anthelmintic widely used in livestock. Further studies should be carried out to assess the practical options of this interaction.

## Figures and Tables

**Table 1:** Non-compartmental pharmacokinetic parameters (mean ± SD) of albendazole sulfoxide (ABZSO) obtained after oral ABZ administration (5 mg/kg) and oral ABZ (5 mg/kg) + intramuscular MEN (10 mg/kg) administration to 12 sheep.

Parameters	ABZ	ABZ + MEN
λ (h^−1^)	0.171 ± 0.063	0.158 ± 0.037
t_1/2λ_ (h)	4.53 ± 1.49	4.60 ± 1.08
AUC_last_ (µg·h/mL)	34.0 ± 8.1	41.3 ± 8.1 ^a^
AUC_0–__∞_ (µg·h/mL)	34.9 ± 8.5	42.2 ± 8.4 ^a^
C_max_ (µg/mL)	1.48 ± 0.32	1.67 ± 0.30 ^a^
t_max_ (h)	10.7 ± 1.6	11.5 ± 2.6
t_lag_ (h)	0.40 ± 0.13	0.27 ± 0.07 ^b^
AUMC_last_ (µg·h^2^/mL)	577.1 ± 183.3	767.9 ± 201.6 ^a^
AUMC_0–__∞_ (µg·h^2^/mL)	622.6 ± 207.3	810.4 ± 218.1
MRT_last_ (h)	16.8 ± 2.2	18.4 ± 2.0 ^a^
MRT_0–__∞_ (h)	17.6 ± 2.3	19.0 ± 2.0

^a^ Significantly different (*t* test, *p* ≤ 0.05); ^b^ Significantly different (Wilcoxon signed-rank test, *p* ≤ 0.05).

**Table 2:** Non-compartmental pharmacokinetic parameters (mean ± SD) of albendazole sulfone (ABZSO_2_) obtained after oral ABZ administration (5 mg/kg) and oral ABZ (5 mg/kg) + intramuscular MEN (10 mg/kg) administration to 12 sheep.

Parameters	ABZ	ABZ + MEN
λ (h^−1^)	0.185 ± 0.059	0.165 ± 0.069
t_1/2λ_ (h)	4.10 ± 1.38	6.67 ± 7.52
AUC_last_ (µg·h/mL)	19.6 ± 3.9	19.1 ± 3.6
AUC_0–__∞_ (µg·h/mL)	19.7 ± 3.9	21.4 ± 8.0
C_max_ (µg/mL)	0.74 ± 0.17	0.72 ± 0.12
t_max_ (h)	29.0 ± 5.0	30.0 ± 3.6
t_lag_ (h)	1.6 ± 0.5	2.0 ± 0.0 ^a^
AUMC_last_ (µg·h^2^/mL)	501.9 ± 138.1	502.3 ± 102.5
AUMC_0–__∞_ (µg·h^2^/mL)	511.6 ± 139.2	693.0 ± 601.9
MRT_last_ (h)	25.4 ± 3.2	26.2 ± 2.0
MRT_0–+__∞_ (h)	25.7 ± 3.2	29.6 ± 9.4

^a^ Significantly different (Wilcoxon signed-rank test, *p* ≤ 0.05).

## Data Availability

Data are available on request the corresponding author.

## Supplementary Materials

The following supporting information can be downloaded at: https://www.mdpi.com/article/10.3390/ani12040463/s1. Figure S1: Representative HPLC chromatogram of plasma sample fortified with ABZ (1 μg/mL), ABZSO (1 μg/mL), ABZSO_2_ (1 μg/mL), and IS (1 μg/mL); Figure S2: Individual plasma concentrations of ABZSO (△ oral ABZ; ○ oral ABZ + intramuscular MEN) and ABZSO_2_ (□ oral ABZ; ♢ oral ABZ + intramuscular MEN) obtained after oral ABZ administration (5 mg/kg) and oral ABZ (5 mg/kg) + intramuscular MEN (10 mg/kg) administration to 12 sheep; Table S1: Data from linear regression analysis of calibration curves; Table S2: Within-run and between-run accuracy for the samples processed; Table S3: Individual and mean ± SD plasma concentrations of ABZSO obtained after oral ABZ administration (5 mg/kg) to 12 sheep; Table S4: Individual and mean ± SD plasma concentrations of ABZSO obtained after oral ABZ (5 mg/kg) + intramuscular MEN (10 mg/kg) administration to 12 sheep; Table S5: Individual and mean ± SD plasma concentrations of ABZSO_2_ obtained after oral ABZ administration (5 mg/kg) to 12 sheep; Table S6: Individual and mean ± SD plasma concentrations of ABZSO_2_ obtained after oral ABZ (5 mg/kg) + intramuscular MEN (10 mg/kg) administration to 12 sheep.

## References

[1] Bongioanni A., Araújo B.S., de Oliveira Y.S., Longhi M.R., Ayala A., Garnero C. (2018). Improving properties of albendazole desmotropes by supramolecular systems with maltodextrin and glutamic acid. AAPS PharmSciTech.

[2] Sawatdee S., Atipairin A., Sae Yoon A., Srichana T., Changsan N., Suwandecha T. (2019). Formulation development of albendazole-loaded self-microemulsifying chewable tablets to enhance dissolution and bioavailability. Pharmaceutics.

[3] Lacey E. (1988). The role of the cytoskeletal protein, tubulin, in the mode of action and mechanism of drug resistance to benzimidazoles. Int. J. Parasitol..

[4] van Genderen P.J.J., van Hellemond J.J., Aronson J.K. (2012). Antihelminthic Drugs. Side Effects of Drugs.

[5] Marriner S.E., Bogan J.A. (1980). Pharmacokinetics of albendazole in sheep. Am. J. Vet. Res..

[6] Benchaoui H.A., Scott E.W., McKellar Q.A. (1993). Pharmacokinetics of albendazole, albendazole sulfoxide and netobimin in goats. J. Vet. Pharmacol. Ther..

[7] Virkel G., Imperiale F., Lifschitz A., Pis A., Alvarez A., Merino G., Prieto J., Lanusse C. (2003). Effect of amphiphilic surfactant agents on the gastrointestinal absorption of albendazole in cattle. Biopharm. Drug Dispos..

[8] Hennessy D.R., Sangster N.C., Steel J.W., Collins G.H. (1993). Comparative pharmacokinetic behaviour of albendazole in sheep and goats. Int. J. Parasitol..

[9] McKellar Q.A., Coop R.L., Jackson F. (1995). The pharmacokinetics of albendazole metabolites following administration of albendazole, albendazole sulfoxide and netobimin to one-month-and eight-month-old sheep. Int. J. Parasitol..

[10] Alvarez L.I., Saumell C.A., Sanchez S.F., Lanusse C.E. (1996). Plasma disposition kinetics of albendazole metabolites in pigs fed different diets. Res. Vet. Sci..

[11] Sanyal P.K. (1998). Effect of single and divided dose administration on the pharmacokinetics of albendazole in sheep and goat. Vet. J..

[12] Sánchez S., Sallovitz J., Savio E., Mckellar Q., Lanusse C. (2000). Comparative availability of two oral dosage forms of albendazole in dogs. Vet. J..

[13] Alvarez L., Lifschitz A., Entrocasso C., Manazza J., Mottier L., Borda B., Virkel G., Lanusse C. (2008). Evaluation of the interaction between ivermectin and albendazole following their combined use in lambs. J. Vet. Pharmacol. Ther..

[14] Suarez G., Alvarez L., Castells D., Moreno L., Fagiolino P., Lanusse C. (2014). Evaluation of pharmacological interactions after administration of a levamisole, albendazole and ivermectin triple combination in lambs. Vet. Parasitol..

[15] Kumbhakar N.K., Sanyal P.K., Rawte D., Kumar D., Kerketta A.E., Pal S. (2016). Efficacy of pharmacokinetic interactions between piperonyl butoxide and albendazole against gastrointestinal nematodiasis in goats. J. Helminthol..

[16] Galtier P., Alvinerie M., Delatour P. (1986). In vitro sulfoxidation of albendazole by ovine liver microsomes: Assay and frequency of various xenobiotics. Am. J. Vet. Res..

[17] Galtier P., Alvinerie M., Plusquellec Y., Tufenkji A.E., Houin G. (1991). Decrease in albendazole sulphonation during experimental fascioliasis in sheep. Xenobiotica.

[18] Delatour P., Cure M.C., Benoit E., Garnier F. (1986). Netobimin (Totabin-SCH): Preliminary investigations on metabolism and pharmacology. J. Vet. Pharmacol. Ther..

[19] Virkel G., Lifschitz A., Sallovitz J., Pis A., Lanusse C. (2004). Comparative hepatic and extrahepatic enantioselective sulfoxidation of albendazole and fenbendazole in sheep and cattle. Drug Metab. Dispos..

[20] Virkel G., Lifschitz A., Pis A., Lanusse C. (2002). In vitro ruminal biotransformation of benzimidazole sulphoxide anthelmintics: Enantioselective sulphoreduction in sheep and cattle. J. Vet. Pharmacol. Ther..

[21] Zhang H., Zhao J., Chen B., Ma Y., Li Z., Shou X., Wen L., Yuan Y., Gao H., Ruan J. (2020). Pharmacokinetics and tissue distribution study of liposomal albendazole in naturally *Echinococcus granulosus* infected sheep by a validated UPLC-Q-TOF-MS method. J. Chromatogr. B.

[22] Lanusse C.E., Prichard R.K. (1992). Methimazole increases the plasma concentrations of the albendazole metabolites of netobimin in sheep. Biopharm. Drug Dispos..

[23] Ali D.N., Hennessy D.R. (1995). The effect of reduced feed intake on the efficacy of oxfendazole against benzimidazole resistant *Haemonchus contortus* and *Trichostrongylus colubriformis* in sheep. Int. J. Parasitol..

[24] Sánchez S.F., Alvarez L.I., Lanusse C.E. (1996). Nutritional condition affects the disposition kinetics of albendazole in cattle. Xenobiotica.

[25] Lifschitz A., Virkel G., Mastromarino M., Lanusse C. (1997). Enhanced plasma availability of the metabolites of albendazole in fasted adult sheep. Vet. Res. Commun..

[26] Sánchez S., Alvarez L., Sallovitz J., Lanusse C. (2000). Enhanced plasma and target tissue availabilities of albendazole and albendazole sulphoxide in fasted calves: Evaluation of different fasting intervals. J. Vet. Pharmacol. Ther..

[27] Mares S.S., Jung C.H., López A.T., González-Esquivel D.F. (2005). Influence of a Mexican diet on the bioavailability of albendazole. Basic Clin. Pharmacol. Toxicol..

[28] Ochoa D., Saiz-Rodríguez M., González-Rojano E., Román M., Sánchez-Rojas S., Wojnicz A., Ruiz-Nuño A., García-Arieta A., Abad-Santos F. (2021). High-fat breakfast increases bioavailability of albendazole compared to low-fat breakfast: Single-dose study in healthy subjects. Front. Pharmacol..

[29] Schmidt L.E., Dalhoff K. (2002). Food-drug interactions. Drugs.

[30] Ceballos L., Krolewiecki A., Juárez M., Moreno L., Schaer F., Alvarez L.I., Cimino R., Walson J., Lanusse C.E. (2018). Assessment of serum pharmacokinetics and urinary excretion of albendazole and its metabolites in human volunteers. PLoS Negl. Trop. Dis..

[31] Jung H., Medina L., García L., Fuentes I., Moreno-Esparza R. (1998). Absorption studies of albendazole and some physicochemical properties of the drug and its metabolite albendazole sulphoxide. J. Pharm. Pharmacol..

[32] Lund J., Lassen J.B. (1969). Elimination and distribution of menbutone (Genabil) in rats. Acta Pharmacol. Toxicol..

[33] Symonds H.W. (1982). The choleretic effect of menbutone and clanobutin sodium in steers. Vet. Rec..

[34] Heads of Medicines Agencies VMRI (Veterinary Mutual Information Recognition) Product Index. https://www.hma.eu/vmriproductindex.html.

[35] (2021). CMDv/GUI/032 Guidance for Link to National Databases of Authorised Products.

[36] Spanish Agency of Medicines and Medical Devices (AEMPS) List of Laboratories Certified for Good Laboratory Practice Compliance [Listado de Laboratorios Certificados para el Cumplimiento de Buenas Prácticas de Laboratorio]. https://www.aemps.gob.es/industria-farmaceutica/buenas-practicas-de-laboratorio/listadolab-bpl/.

[37] (2012). Guideline on Bioanalytical Method Validation EMEA/CHMP/EWP/192217/2009.

[38] Yamaoka K., Nakagawa T., Uno T. (1978). Statistical moments in pharmacokinetics. J. Pharmacokinet. Biopharm..

[39] Gibaldi M., Perrier D. (1982). Pharmacokinetics.

[40] Wagner J.D. (1993). Pharmacokinetics for the Pharmaceutical Scientist.

[41] Delatour P., Benoit E., Caude M., Tambute A. (1990). Species differences in the generation of the chiral sulfoxide metabolite of albendazole in sheep and rats. Chirality.

[42] Hennessy D.R., Steel J.W., Lacey E., Eagleson G.K., Prichard R.K. (1989). The disposition of albendazole in sheep. J. Vet. Pharmacol. Ther..

[43] Lanusse C.E., Gascon L.H., Prichard R.K. (1995). Comparative plasma disposition kinetics of albendazole, fenbendazole, oxfendazole and their metabolites in adult sheep. J. Vet. Pharmacol. Ther..

[44] Merino G., Alvarez A.I., Redondo P.A., Garcia J.L., Larrodé O.M., Prieto J.G. (1999). Bioavailability of albendazole sulphoxide after netobimin administration in sheep: Effects of fenbendazole coadministration. Res. Vet. Sci..

[45] Alvarez L., Suárez G., Ceballos L., Moreno L., Lanusse C. (2012). Dose-dependent systemic exposure of albendazole metabolites in lambs. J. Vet. Pharmacol. Ther..

[46] Swarnkar C.P., Sanyal P.K., Singh D., Khan F.A., Bhagwan P.S. (1998). Comparative disposition kinetics of albendazole in sheep following oral and intraruminal administration. Vet. Res. Commun..

[47] Delatour P., Benoit E., Garnier F., Besse S. (1990). Chirality of the sulphoxide metabolites of fenbendazole and albendazole in sheep. J. Vet. Pharmacol. Ther..

[48] Delatour P., Garnier F., Benoit E., Caude I. (1991). Chiral behaviour of the metabolite albendazole sulphoxide in sheep, goats and cattle. Res. Vet. Sci..

[49] Lanusse C.E., Gascon L.H., Prichard R.K. (1993). Gastrointestinal distribution of albendazole metabolites following netobimin administration to cattle: Relationship with plasma disposition kinetics. J. Vet. Pharmacol. Ther..

[50] Lanusse C.E., Prichard R.K. (1993). Clinical pharmacokinetics and metabolism of benzimidazole anthelmintics in ruminants. Drug Metab. Rev..

[51] Miró M.V., Silva C.R.E., Viviani P., Luque S., Lloberas M., Costa-Júnior L.M., Lanusse C., Virkel G., Lifschitz A. (2020). Combination of bioactive phytochemicals and synthetic anthelmintics: In vivo and in vitro assessment of the albendazole-thymol association. Vet. Parasitol..

[52] Redondo P., Alvarez A., García J., Villaverde C., Prieto J. (1998). Influence of surfactants on oral bioavailability of albendazole based on the formation of the sulphoxide metabolites in rats. Biopharm. Drug Dispos..

[53] del Estal J., Alvarez A., Villaverde C., Coronel P., Fabra S., Prieto J. (1991). Effect of surfactants on albendazole absorption. J. Pharm. Biomed. Anal..

[54] (2010). European Commission Commission Regulation (EU) N^o^ 37/2010 of 22 December 2009 on pharmacologically active substances and their classification regarding maximum residue limits in foodstuffs of animal origin. Off. J. Eur. Union.

