# Supplementary material for: Improvement of Albendazole Bioavailability with Menbutone Administration in Sheep
Source: Animals (Basel). 2022 Feb 14;12(4):463. doi: 10.3390/ani12040463 (PMC8868263; doi:10.3390/ani12040463)
Supplement: Supplementary file 1 [file animals-12-00463-s001.zip › animals-1545204-supplementary.pdf]

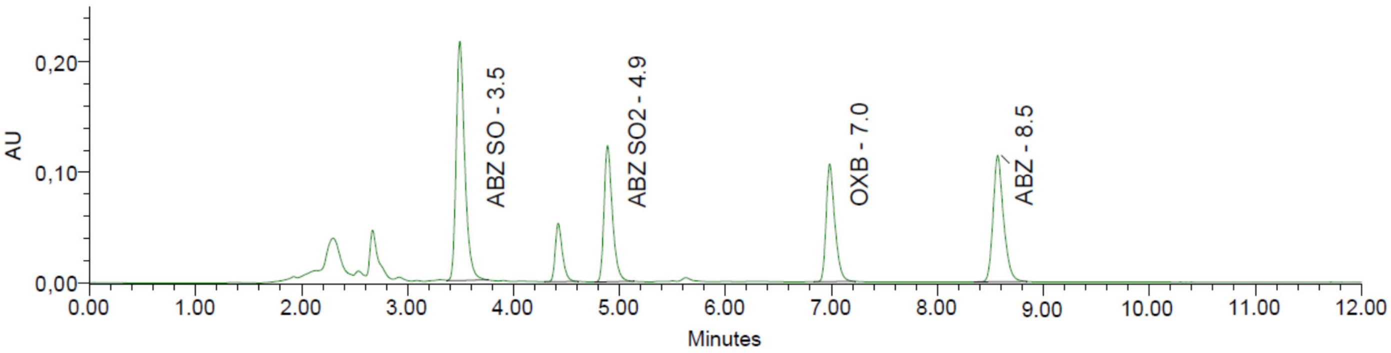

Supplementary Figure S1. Representative HPLC chromatogram of plasma sample fortified with ABZ (1 μg/mL), ABZSO (1 μg/mL), $ABZSO_2$ (1 μg/mL), and IS (1 μg/mL).

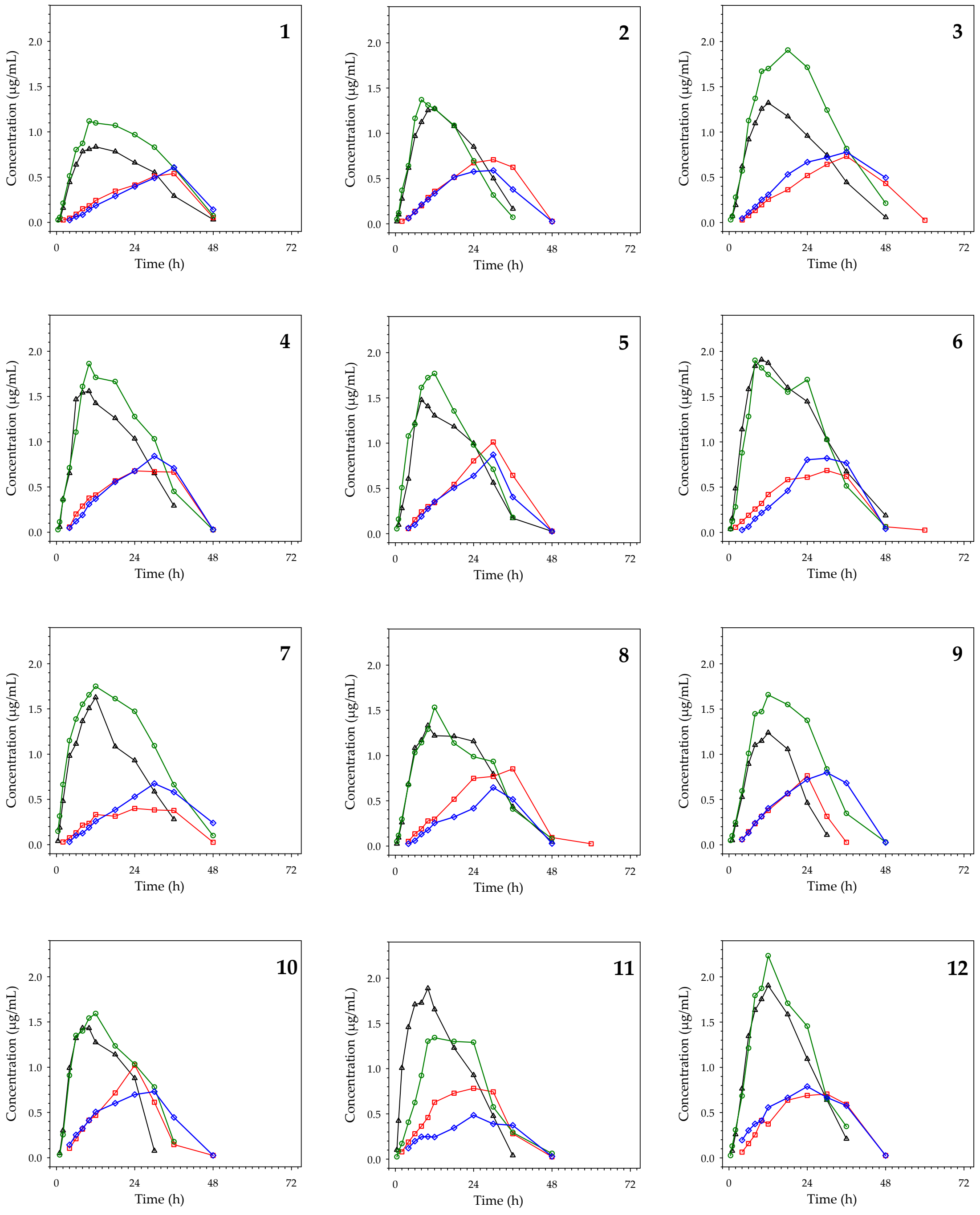

Supplementary Figure S2. Individual plasma concentrations of ABZSO (△ oral ABZ; ○ oral ABZ + intramuscular MEN) and $ABZSO_2$ (□ oral ABZ; ◊ oral ABZ + intramuscular MEN) obtained after oral ABZ administration (5 mg/kg) and oral ABZ (5 mg/kg) + intramuscular MEN (10 mg/kg) administration to 12 sheep.

Supplementary Table S1. Data from linear regression analysis of calibration curves.

Characteristic	ABZ	ABZSO	$ABZSO_2$
Curve 1 (R^2)	y = 0.645 x + 0.014 (0.995)	y = 1.030 x + 0.041 (0.999)	y = 0.554 x + 0.006 (0.999)
Curve 2 (R^2)	y = 1.378 x + 0.013 (0.999)	y = 1.849 x + 0.009 (0.999)	y = 1.288 x – 0.007 (0.999)
Curve 3 (R^2)	y = 1.408 x + 0.012 (0.999)	y = 1.923 x + 0.016 (0.999)	y = 1.356 x – 0.003 (0.999)
LLOQ (µg/mL)	0.025	0.025	0.025
LOD (µg/mL)	0.001	0.002	0.007
Recovery (%) (Mean ± SD)	95.4 ± 7.2	100.1 ± 6.4	99.5 ± 7.2

Supplementary Table S2. Within-run and between-run accuracy for the samples processed.

Compound	Nominal concentration (µg/mL)	Accuracy (% from nominal concentration)		Precision (% CV)	
		Within-run (range)	Between-run	Within-run (range)	Between-run
ABZ	0.025	84.7-107.2	95.7	4.3–10.4	6.8
	0.075	86.0-96.4	92.6	2.1-4.2	3.1
	1	100.4-106.0	102.3	1.9-2.4	2.1
	1.5	92.0-110.3	99.9	2.1-5.9	3.9
ABZSO	0.025	86.1-115.9	99.0	4.4-8.6	5.9
	0.075	89.3-103.2	95.5	3.4-5.8	4.7
	1	97.9-112.9	103.0	2.1-6.4	3.8
	1.5	98.9-112.8	103.5	2.8–6.5	4.1
$ABZSO_2$	0.025	87.0-111.6	99.9	3.8-6.6	5.1
	0.075	97.3-102.8	99.9	0.7–1.6	1.3
	1	87.5-110.5	101.2	4.1-6.2	4.9
	1.5	87.0-112.2	99.8	1.3–5.1	3.8

Supplementary Table S3. Individual and mean ± SD plasma concentrations of ABZSO obtained after oral ABZ administration (5 mg/kg) to 12 sheep.

**Time**	**Concentrations** (µg/mL)												
(h)	**1**	**2**	**3**	**4**	**5**	**6**	**7**	**8**	**9**	**10**	**11**	**12**	**Mean ± SD**
0.25													
0.5		0.025				0.036	0.040	0.025			0.101		0.019 ± 0.030
1	0.027	0.100	0.071	0.059	0.097	0.151	0.186	0.092	0.048	0.050	0.423	0.077	0.115 ± 0.107
2	0.159	0.276	0.191	0.367	0.280	0.484	0.482	0.261	0.221	0.300	1.007	0.259	0.357 ± 0.229
4	0.444	0.618	0.618	0.652	0.606	1.138	0.982	0.686	0.527	0.992	1.459	0.764	0.790 ± 0.295
6	0.637	0.968	0.918	1.468	1.222	1.582	1.114	1.082	0.894	1.321	1.709	1.345	1.188 ± 0.313
8	0.784	1.123	1.095	1.543	1.477	1.838	1.365	1.169	1.103	1.432	1.728	1.634	1.358 ± 0.309
10	0.809	1.253	1.257	1.558	1.407	1.908	1.507	1.331	1.148	1.431	1.887	1.753	1.437 ± 0.316
12	0.834	1.270	1.321	1.426	1.304	1.871	1.628	1.220	1.239	1.274	1.654	1.904	1.412 ± 0.304
18	0.783	1.079	1.171	1.260	1.183	1.600	1.084	1.212	1.057	1.141	1.230	1.586	1.199 ± 0.222
24	0.660	0.851	0.958	1.034	0.999	1.446	0.931	1.158	0.464	0.879	0.927	1.093	0.950 ± 0.244
30	0.551	0.500	0.743	0.651	0.562	1.026	0.586	0.795	0.109	0.076	0.477	0.640	0.560 ± 0.265
36	0.291	0.164	0.443	0.293	0.170	0.673	0.281	0.434			0.041	0.210	0.250 ± 0.200
48	0.031		0.057		0.025	0.186		0.047					0.029 ± 0.054
60													0.053 ± 0.061
72													

SD: standard deviation.

Supplementary Table S4. Individual and mean ± SD plasma concentrations of ABZSO obtained after oral ABZ (5 mg/kg) + intramuscular MEN (10 mg/kg) administration to 12 sheep.

**Time**	**Concentrations** (μg/mL)												
(h)	**1**	**2**	**3**	**4**	**5**	**6**	**7**	**8**	**9**	**10**	**11**	**12**	**Mean ± SD**
0.25													
0.5	0.025	0.047	0.027	0.026	0.052	0.035	0.147	0.047	0.045		0.023	0.023	0.042 ± 0.036
1	0.052	0.118	0.066	0.112	0.157	0.119	0.315	0.115	0.097	0.030	0.087	0.128	0.116 ± 0.072
2	0.209	0.368	0.278	0.355	0.506	0.279	0.662	0.296	0.243	0.252	0.171	0.307	0.327 ± 0.136
4	0.512	0.640	0.569	0.711	1.077	0.876	1.148	0.676	0.594	0.908	0.406	0.682	0.733 ± 0.226
6	0.802	1.164	1.124	1.104	1.207	1.279	1.386	1.031	1.009	1.350	0.623	1.211	1.107 ± 0.220
8	0.871	1.370	1.370	1.609	1.614	1.900	1.550	1.141	1.446	1.399	0.922	1.792	1.415 ± 0.315
10	1.119	1.310	1.670	1.862	1.722	1.817	1.654	1.290	1.469	1.541	1.302	1.871	1.552 ± 0.253
12	1.097	1.271	1.700	1.709	1.768	1.744	1.749	1.532	1.657	1.592	1.341	2.233	1.616 ± 0.290
18	1.070	1.086	1.904	1.664	1.354	1.549	1.612	1.137	1.549	1.235	1.300	1.707	1.431 ± 0.272
24	0.968	0.693	1.714	1.277	0.978	1.687	1.472	0.987	1.374	1.033	1.290	1.454	1.244 ± 0.315
30	0.830	0.315	1.240	1.031	0.709	1.023	1.092	0.933	0.837	0.780	0.575	0.649	0.835 ± 0.253
36	0.600	0.070	0.816	0.449	0.178	0.512	0.662	0.407	0.345	0.176	0.294	0.345	0.404 ± 0.218
48	0.073		0.211	0.025		0.061	0.098	0.081	0.029		0.061		
60													
72													

SD: standard deviation.

Supplementary Table S5. Individual and mean ± SD plasma concentrations of $ABZSO_2$ obtained after oral ABZ administration (5 mg/kg) to 12 sheep.

Time	Concentrations (µg/mL)												
(h)	**1**	**2**	**3**	**4**	**5**	**6**	**7**	**8**	**9**	**10**	**11**	**12**	**Mean ± SD**
0.25													
0.5													
1													
2	0.027	0.027				0.056	0.029				0.079		0.018 ± 0.025
4	0.046	0.066	0.027	0.057	0.055	0.121	0.076	0.051	0.058	0.103	0.188	0.062	0.076 ± 0.042
6	0.090	0.138	0.075	0.202	0.153	0.188	0.131	0.135	0.145	0.209	0.280	0.158	0.159 ± 0.053
8	0.150	0.199	0.132	0.288	0.240	0.257	0.216	0.190	0.232	0.316	0.363	0.254	0.236 ± 0.063
10	0.182	0.290	0.196	0.378	0.299	0.319	0.235	0.278	0.312	0.414	0.460	0.412	0.315 ± 0.084
12	0.242	0.359	0.256	0.411	0.343	0.418	0.333	0.297	0.379	0.468	0.628	0.374	0.376 ± 0.099
18	0.345	0.514	0.363	0.568	0.544	0.583	0.314	0.517	0.563	0.716	0.728	0.637	0.533 ± 0.129
24	0.413	0.673	0.518	0.678	0.803	0.608	0.400	0.749	0.762	1.022	0.783	0.689	0.675 ± 0.167
30	0.510	0.707	0.643	0.669	1.012	0.683	0.383	0.770	0.314	0.613	0.743	0.705	0.646 ± 0.175
36	0.538	0.625	0.732	0.664	0.644	0.618	0.378	0.854	0.027	0.144	0.280	0.593	0.508 ± 0.238
48	0.052	0.027	0.431	0.028	0.027	0.061	0.027	0.093		0.025	0.025	0.025	0.068 ± 0.112
60			0.025			0.025		0.025					0.006 ± 0.011
72													

SD: standard deviation.

Supplementary Table S6. Individual and mean ± SD plasma concentrations of $ABZSO_2$ obtained after oral ABZ (5 mg/kg) + intramuscular MEN (10 mg/kg) administration to 12 sheep.

**Time**	**Concentrations** (µg/mL)												
(h)	**1**	**2**	**3**	**4**	**5**	**6**	**7**	**8**	**9**	**10**	**11**	**12**	**Mean ± SD**
0.25													
0.5													
1													
2													
4	0.025	0.060	0.045	0.047	0.062	0.027	0.030	0.025	0.058	0.142	0.120	0.195	0.070 ± 0.054
6	0.063	0.131	0.111	0.121	0.097	0.063	0.102	0.058	0.132	0.252	0.198	0.303	0.136 ± 0.077
8	0.083	0.212	0.173	0.191	0.189	0.150	0.126	0.130	0.239	0.322	0.246	0.375	0.203 ± 0.083
10	0.142	0.268	0.253	0.310	0.275	0.216	0.189	0.176	0.313	0.413	0.248	0.410	0.268 ± 0.085
12	0.186	0.336	0.307	0.370	0.354	0.272	0.258	0.255	0.403	0.505	0.244	0.557	0.337 ± 0.110
18	0.290	0.516	0.531	0.555	0.506	0.460	0.384	0.322	0.569	0.601	0.345	0.664	0.479 ± 0.119
24	0.395	0.577	0.666	0.676	0.639	0.802	0.530	0.417	0.723	0.698	0.485	0.789	0.616 ± 0.136
30	0.491	0.589	0.717	0.842	0.873	0.820	0.676	0.647	0.798	0.730	0.388	0.666	0.686 ± 0.145
36	0.608	0.379	0.778	0.708	0.404	0.767	0.581	0.516	0.682	0.445	0.373	0.577	0.568 ± 0.147
48	0.140	0.025	0.494	0.029	0.025	0.039	0.239	0.028	0.025	0.025	0.030	0.025	0.094 ± 0.142
60													
72													

SD: standard deviation.
